# SIX1 Predicts Poor Prognosis and Facilitates the Progression of Non-small Lung Cancer via Activating the Notch Signaling Pathway

**DOI:** 10.7150/jca.61385

**Published:** 2022-01-01

**Authors:** Shanshan Huang, Wanling Lin, Lei Wang, Yuan Gao, Xun Yuan, Peng Zhang, Yuan Chen, Qian Chu

**Affiliations:** Department of Oncology, Tongji Hospital, Tongji Medical College, Huazhong University of Science and Technology, Wuhan, China.

**Keywords:** SIX1, non-small cell lung cancer, Notch signaling, epithelial-mesenchymal transition.

## Abstract

**Background:** Many transcription factors involved in embryonic development and reactivated in tumors are considered potential prognostic biomarkers and novel therapeutic targets in various cancers. Sine oculis homeobox homolog 1 (SIX1), a developmentally restricted transcriptional regulator, plays a critical role during tumor initiation and development. However, the prognostic value and biological function of SIX1 in non-small cell lung cancer (NSCLC) remain unclear.

**Methods:** Bioinformatic analyses were conducted to investigate the expression of *SIX1* in cancer and adjacent normal tissues of NSCLC and further explore the correlations between *SIX1* expression and clinical outcomes. Western blotting and RT-PCR analysis were performed to detect of *SIX1* expression level in NSCLC cell lines and normal bronchial epithelial cell. EdU, CCK-8, clonal formation assay, wound healing and transwell assay were performed to explore the effects of gain- or loss-of-function of SIX1 on cellular proliferation, migration and invasion *in vitro*. Gene set enrichment analysis (GSEA) was used to identify the potential signaling pathways involved in SIX1 mediated biological function and the correlation was confirmed by western blotting and RT-PCR analysis. *In vivo* experiment was conducted to further validate the tumor-promoting effects of SIX1.

**Results:** Bioinformatic analysis indicated that *SIX1* was markedly upregulated in NSCLC tissues of and positively correlated with poor prognosis of patients with NSCLC. Ectopic expression of SIX1 facilitated proliferation, migration, invasion, and epithelial-mesenchymal transition (EMT) of NSCLC cells. On the contrary, knocking down SIX1 exhibited the opposite effects. Mechanistic studies suggested that SIX1 activated the Notch pathway to promote the malignant biological behaviors of NSCLC, which could be reversed by inhibiting the Notch signaling with γ-secretase inhibitor.

**Conclusions:** SIX1 could facilitate multiple malignant biological behaviors by activating the Notch signaling pathway and function as a promising prognostic biomarker.

## Introduction

Lung cancer is the leading cause of cancer-related death worldwide. According to the latest data from Global Cancer Statistics, there were approximately 2.2 million new cases and 1.8 million deaths of lung cancer in 2020, accounting for approximately 1 in 10 (11.4%) cancers diagnosed and 1 in 5 (18.0%) deaths 1. Approximately 85% of lung cancers are diagnosed as non-small cell lung cancer (NSCLC), including lung adenocarcinoma (LUAD), lung squamous cell carcinoma (LUSC), and large cell lung cancer (LCLC), etc 2. Despite the rapid development of advanced diagnostic methods and precise individual therapeutic regimens, uncontrolled proliferation and metastasis still lead to poor prognosis of patients with NSCLC. Therefore, an in-depth understanding of the molecular mechanisms involved in tumor progression is crucial for developing novel therapies for patients with NSCLC.

Aberrant activation of developmental genes in mature tissues is one of the important causes of tumorigenesis. Certainly, many homeobox genes, which were known to be critical for specific cells development, displayed deregulated expression in cancer 3-5. Evidences have suggested that their inappropriate activation played a complex causal role in carcinogenesis and tumor progression 6. Homeobox transcription factor *SIX1*, which belongs to the *SIX* families, is essential for the development of many organs, including the eye, ear, muscle and kidney 7, 8. In recent decades, SIX1 has been reported to be overexpressed in numerous human cancers, such as breast cancer 9, 10, ovarian cancer 11, colorectal cancer 12, thyroid carcinoma 13, and prostate cancer 14, leading to a more aggressive cell phenotype and poorer prognosis. Mimae T et al. showed that SIX1 was 136-fold upregulated in microinvasion cancer cells compared to lepidic growth cancer cells in minimally invasive adenocarcinomas using a laser capture microdissection system 15, suggesting that SIX1 may be a reliable progressive biomarker in NSCLC. However, the prognostic value and biological function of SIX1 in NSCLC required further investigation.

In this study, we revealed that *SIX1* level was significantly elevated in NSCLC, and the high level of *SIX1* was correlated with shortened time to relapse and decreased overall survival (OS). Overexpression of SIX1 could promote proliferation, migration, invasion, and epithelial-mesenchymal transition (EMT) of NSCLC cells, whereas knockdown of SIX1 exhibited the opposite effects. In addition, we found that SIX1 could activate the Notch signaling pathway in NSCLC, and inhibition of the Notch signaling pathway with γ-secretase inhibitor could reverse a series of SIX1-mediated malignant phenotypes. In summary, our results indicated that SIX1 might serve as a potential prognostic biomarker for NSCLC, and suggested that the SIX1/Notch axis might be a promising target for combating NSCLC progression.

## Materials and Methods

### Data mining and analysis

The TIMER database (http://timer.cistrome.org/) was used to analyze the mRNA expression of *SIX1* in pan-cancer. The Oncomine database (http://www.oncomine.org) was used to further analyze the mRNA levels of* SIX1* in cancer tissues and adjacent normal tissues of NSCLC. The UALCAN database (http://ualcan.path.uab.edu/) exhibited comprehensive cancer transcriptome and clinical patient data (pulled from TCGA). GSE31210 was downloaded from the GEO database to analyze the correlation between the level of SIX1 and prognosis. The potential related signaling pathway was detected through gene set enrichment analysis (GSEA).

### Cell Cultures and reagents

The human NSCLC cell lines A549, H1299, PC-9, H460 and the normal bronchial epithelial cell (HBE) were obtained from the oncology laboratory of Tongji Hospital, Wuhan, China. All cells were cultured in RPMI 1640 medium (HyClone, Logan, UT) supplemented with 10% fetal bovine serum (FBS) (Life Technologies, Grand Island, NY), and then stored in a 37 °C humidified incubator with 5% CO2. The γ-secretase inhibitor DAPT was purchased from MedChemExpress (Monmouth Junction, NJ).

### Western blotting analysis

Cells were lysed with RIPA lysis buffer (Beyotime, Shanghai, China) on ice for half an hour. The supernatant was collected after centrifugation at 12 000 g at 4℃ for 20 min. Protein concentration was measured by the BCA protein assay kit (Beyotime, Shanghai, China). The denatured proteins were separated by SDS‐PAGE gels and transferred to the 0.45 μm PVDF membranes (Millipore, Billerica, USA). The membranes were blocked with 5% BSA for 1 h at room temperature. Next, the membranes were incubated with primary antibodies including SIX1 (#16960), E-cadherin (#3195), Vimentin (#5741), Notch1 (#4380), Hes1 (#11988), Notch2 (#5732), (1:1000, Cell Signaling Technology, Beverly, MA, USA), Tubulin mouse monoclonal antibody (#66031‐1‐Ig), (1:5000, Proteintech, Wuhan, China) at 4°C overnight, and then incubated with corresponding secondary antibodies (1:5000, Boster, Wuhan, China) for 1 hour at room temperature. Finally, West Pico Plus Chemiluminescent Substrate (Thermo Scientific, Marietta, OH, USA) was used to detect the indicated proteins. Image J (National Institutes of Health, Bethesda, MA, USA) was used to analyze the intensity of the blotting bands.

### Real-time PCR (RT-PCR) analysis

Total RNA was extracted from cells with TRIzol reagent (Takara, Shiga, Japan) and then reverse transcribed into cDNA using a PrimeScript RT-PCR kit (Takara, Japan). Real-time PCR analysis was conducted using SYBR Premix Ex Taq (Takara, Japan) with a 7900 Real-time PCR system (Applied Biosystems, USA). The △△CT method was adopted to calculate the relative mRNA expression of indicated genes, which was further normalized to the *β-actin* mRNA levels. The sequences of the primers were shown in Table 1.

### Cell transfection with lentivirus

Cells were cultured overnight to 60% confluence in RPMI-1640 containing 10% FBS before transfection. Subsequently, the cell culture medium was replaced with a serum-free medium containing the lentiviruses (GeneChem, Shanghai, China) at a multiplicity of infection of 20 and polybrene (GeneChem) at a final concentration of 5 μg/ml. Cells as negative control were transduced with corresponding empty vector lentiviruses in the same manner. According to the cell status, the cells were transferred to a complete medium at 12-24h after transfection and further cultured for another 48 h. Then cells were screened with 2 μg/ml puromycin for one week.

### Cell transfection with siRNA

Transfection with siRNA was performed with Lipofectamine 2000 (Invitrogen) according to the instructions. The small interfering RNAs used to knockdown SIX1 were:

siSIX1-1:

Sense, GCUACUGCUUCAAGGAGAATT,

Antisense, UUCUCCUUGAAGCAGUAGCTT,

siSIX1-2:

Sense, GCGACCACCUGCACAAGAATT,

Antisense, UUGUUGUGCAGGUGGUCGCTT,

siSIX1-3:

Sense, GUGCGAGGUUCUGCAGCAATT,

Antisense, UUGCUGCAGAACCUCGCACTT.

### Immunofluorescence staining

A total of 3 × 10^4^ cells were seeded in 24-well plates and incubated at 37 °C overnight. Cells were washed with PBS and then fixed with 4% polyformaldehyde for 15 min. Next, cells were permeabilized with 0.5% Triton X-100 for 20 min and blocked with 5% BSA for 30 min at room temperature. After blocking, cells were incubated with diluted primary antibodies (SIX1, 1:400, #16960, Vimentin, 1:400, #5741) at 4 °C overnight. The next day, cells were incubated with Alexa Fluor Cy3-conjugated secondary antibody (1:200, Promoter, Wuhan, China) at room temperature for 1 h and then incubated with DAPI for 5 min. Finally, the cells were covered with anti-fluorescence quenching agents and photographed under a fluorescence microscope. Quantification of fluorescence intensities was conducted using Image J software. The exposure times and microscope settings were consistent for each captured images. Three random areas were selected to take three images in each independent experiment, and three independent experiments were repeated.

### Cell counting kit‐8 (CCK8) assay

The transfected cells were plated into 96-well plates with 2 × 10^3^ cells/100 μl per well. After the cells attached to the well, all cells incubated for another 0, 24, 48, or 72 hours. Then, 100ul serum-free medium containing 10% CCK8 was added to each well and incubated for another 2 h. The absorbance was measured at 450 nm via a Power Wave XS microplate reader (BioTek, Winooski, VT, USA). Each experiment was conducted at least three times.

### EdU assay

The transfected cells were plated into 96-well plates with 1 × 10^4^ cells/100 μl per well. After the cells were attached to the well, various reagents were added sequentially according to the EdU kit's instructions (RiboBio, Guangzhou, China). Edu positive cells were observed under the fluorescence microscope.

### Transwell assays

Transwell 24‐well plates with an 8‐μm diameter filter (Corning, Corning, NY, USA) were used for the experiments. For the transwell invasion assay, the filters were precoated with 50 μl of diluted extracellular matrix (ECM) gel overnight. 5 × 10^4^ cells (migration) and 1 × 10^5^ cells (invasion) in 200 μl serum‐free RPMI‐1640 medium were placed in the upper chamber and 700 μl RPMI‐1640 medium with 20% FBS was placed in the lower chamber. The plates were then cultured for another 24 hours or 48 hours. Next, the upper cells were fixed with 4% paraformaldehyde and stained with 0.3% crystal violet for 15 min. Finally, the cells passing through the aperture were examined and counted under the microscope.

### Immunohistochemistry (IHC)

At the endpoint of the observation, the tumor tissues were removed and then fixed in 4% paraformaldehyde. The fixed tissues were then embedded in paraffin and sectioned at 4μm thickness. After that, the sections were deparaffinized, rehydrated, and stained using the primary antibodies (SIX1, 1:100, CST; Hes1, 1:2000, CST; Vimentin, 1:200, CST; Ki-67, 1:400, Abcam) at 4 °C overnight. The next day, sections were incubated with the corresponding secondary antibody at 37 °C for 30 min. Finally, images were captured under the microscope. The protein expression was evaluated according to the staining intensity and percentage of positive cells by using an H-score method 16.

### Animal experiments

Five‐week‐old male BALB/c nude mice were used for the construction of the xenograft model *in vivo*. Cells (5×10^^6^/125 μL PBS) were injected subcutaneously into the right flanks of each mouse (five mice per group). Tumor volumes were measured in two dimensions every three days using digital calipers and calculated using the following formula: tumor volume (mm^3^) = 1/2 (length (mm) × (width (mm))^2^). After 24 days of injection, the mice were sacrificed, and the tumor tissues were fixed and embedded for further analyses.

### Statistical analysis

All quantitative data are presented as the mean values ± SD from at least three independent experiments and statistical analyses were undertaken by GraphPad 8.0. The survival analysis was performed using GraphPad 8.0. Significant differences were determined by a two-tailed Student's t-test or one-way ANOVA analysis. Pearson's correlation was used to measure the strength of association between two variables. *p <* 0.05 was considered significant. *p< 0.05, **p < 0.01, ***p < 0.001.

## Results

### Elevated mRNA expression of *SIX1* in many types of cancer

**Figure 1:**
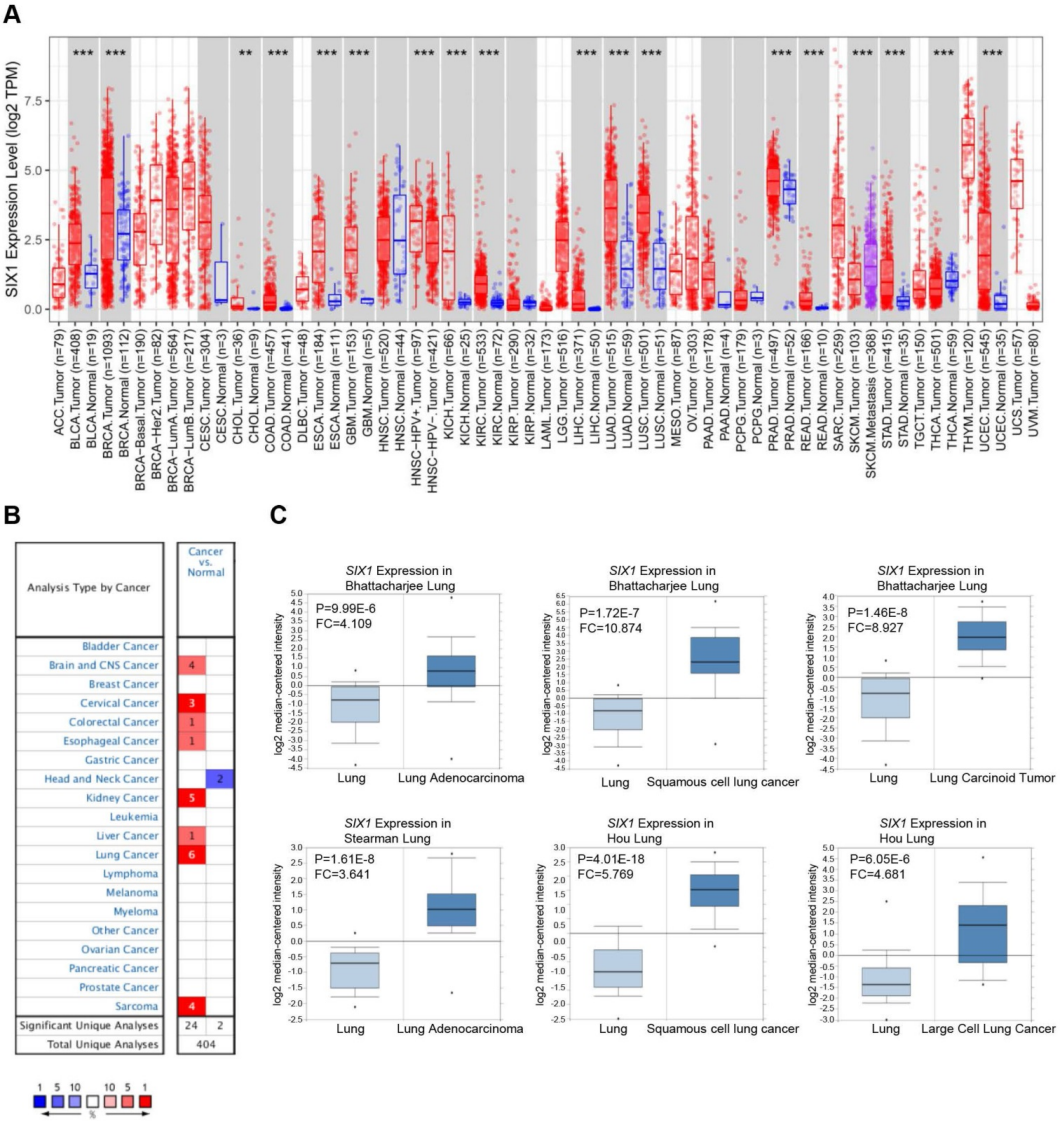
***SIX1* mRNA expression in pan-cancer.** (A) *SIX1* expression in multiple cancers were analyzed using the TIMER database. (B) *SIX*1 expression in multiple cancers were analyzed using the Oncomine database. (C) *SIX1* expression was increased in LUAD, LUSC, lung carcinoid tumor and LCLC tumor tissues. *p< 0.05, **p < 0.01, ***p < 0.001.

First, the expression of *SIX1* in cancers and matched adjacent normal tissues were compared using the TIMER database. The results suggested that *SIX1* mRNA levels were remarkably upregulated in many cancer tissues (BLCA, BRCA, CHOL, COAD, ESCA, GBM, KICH, KIRC, LIHC, LUAD, LUSC, PRAD, READ, STAD, and LUEC), compared with the corresponding adjacent normal tissues. Moreover, SKCM metastatic tissues showed higher *SIX1* mRNA level than SKCM primary tumor tissues (Fig. 1A). We also examined the transcriptional level of *SIX1* in a variety of malignancies using the Oncomine database. The database contains 26 significant, unique analyses. In 24 of the 26 unique analyses, *SIX1* expression was elevated in cancer tissues than in normal tissues, including 6 analyses related to lung cancer (Fig. 1B). In these 6 analyses of lung cancer from Bhattacharjee al., Stesrman et al. and Hou et al., *SIX1* expression levels in NSCLC, including LUAD, LUSC, lung carcinoid tumor, and LCLC, were significantly upregulated in cancer tissues (Fig. 1C).

### *SIX1* predicted poor clinical outcomes in NSCLC

**Figure 2:**
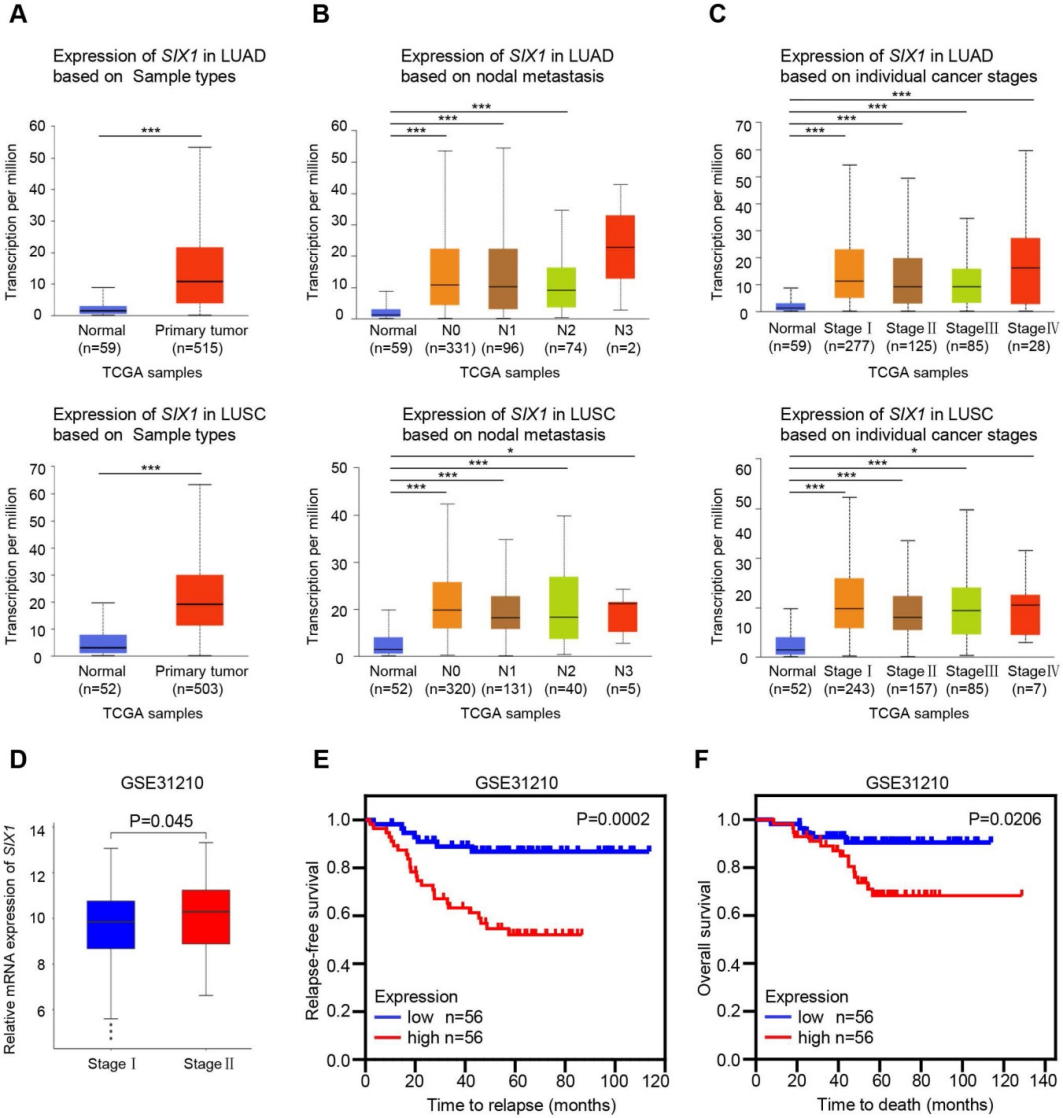
** Correlation of *SIX1* expression and clinicopathological features in NSCLC patients.** (A) *SIX1* was significantly upregulated in LUAD and LUSC in the UALCAN database. (B) *SIX1* expression in LUAD and LUSC with different lymph node metastatic states. (C) *SIX1* expression in LUAD and LUSC with stage Ⅰ to stage Ⅳ diseases. (D) SIX1 expression in patients with different stages in the GSE31210 database. (E) The correlation between SIX1 expression and RFS in NSCLC patients. (F) The correlation between SIX1 expression and OS in NSCLC patients. *p< 0.05, **p < 0.01, ***p < 0.001.

The UALCAN and GEO databases were used to further analyze the *SIX1* expression in NSCLC. Consistently, TCGA-LUAD and TCGA-LUSC samples in the UALCAN database also showed elevated mRNA level of *SIX1* in lung cancer tissues (Fig. 2A). Although there was no significant correlation between the *SIX1* expression and lymph node metastasis or tumor-node-metastasis (TNM) stage when N0, N1, N2, and N3, or stage I, II, III, and IV were compared, the expression of *SIX1* was markedly higher in patients with TCGA-LUAD and TCGA-LUSC than normal controls in a subgroup analysis based on nodal metastasis and disease stages, (Fig. 2B and 2C). In addition, a data mining based on the GSE31210, a database that included 226 primary LUAD of pathological stage I-II, showed that the* SIX1* expression was higher in patients in stage II than in stage I (Fig. 2D). Moreover, survival analysis revealed that high mRNA expression of *SIX1* was associated with the short time to relapse and poor OS in NSCLC (Fig. 2E and 2F). These data suggested that *SIX1* might be a potential prognostic indicator in NSCLC.

### SIX1 promoted proliferation of NSCLC cells

**Figure 3:**
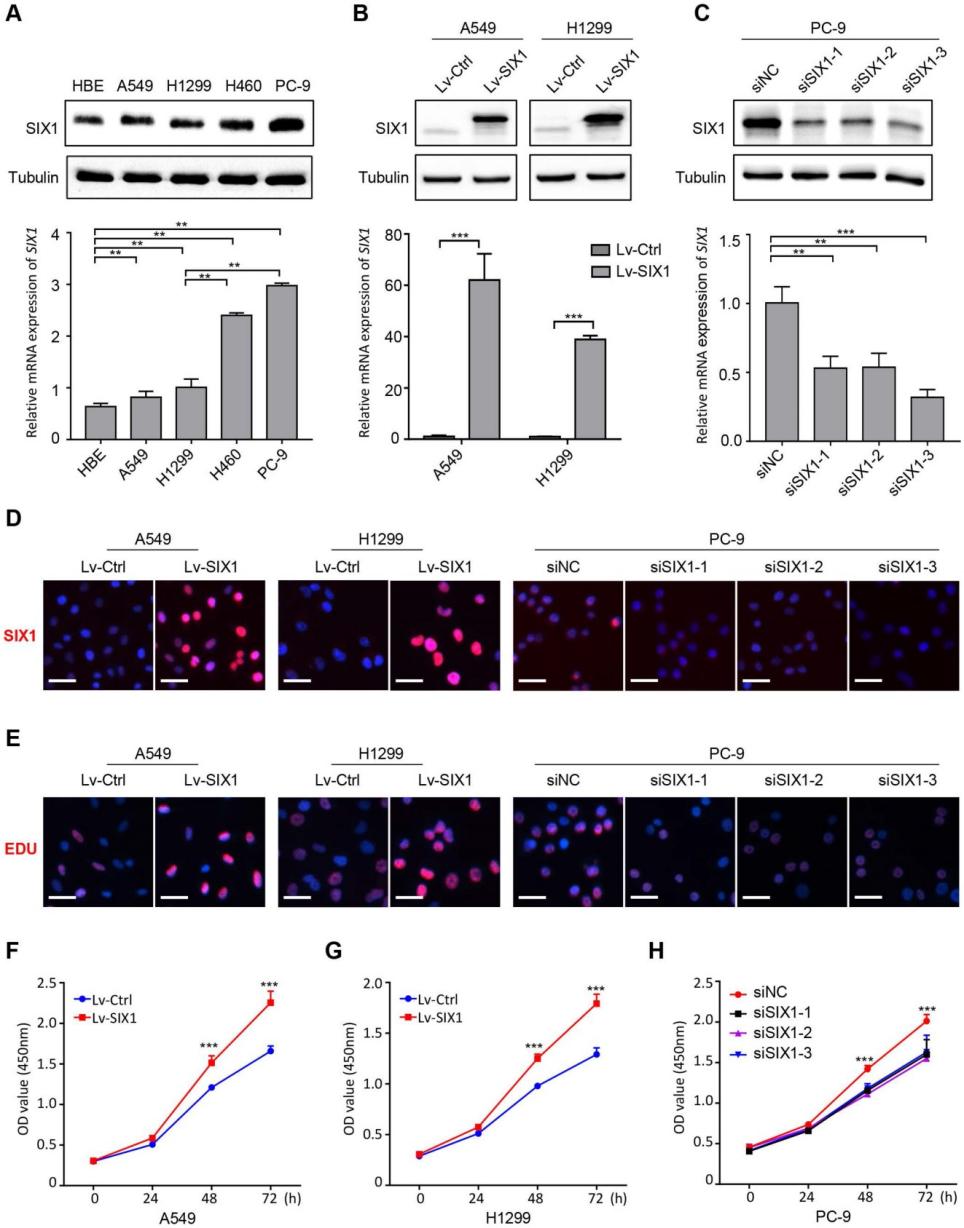
** SIX1 facilitates NSCLC cells growth *in vitro*.** (A) The protein and mRNA level of SIX1 on baseline in four NSCLC cells and one normal bronchial epithelial cell. (B) SIX1 expression in A549 and H1299 cells transfected with SIX1-overexpressing lentivirus. (C) SIX1 expression in PC-9 cells transfected with SIX1 siRNA. (D) Immunofluorescence staining of SIX1 in indicated cells. (E) DNA synthesis in indicated cells by EdU assays. (F) (G) (H) The growth curves of indicated cells by CCK-8 assays. *p< 0.05, **p < 0.01, ***p < 0.001. Graphs show mean ± SD.

Based on the above results, we then conducted a series of gain-of-function and loss-of-function experiments in NSCLC cells to explore the potential role of SIX1 in NSCLC. The endogenous protein and mRNA expression of SIX1 in four NSCLC cell lines (A549, H1299, H460, and PC-9) and a normal human bronchial epithelial cell HBE were measured by western blotting and RT-PCR. Consistent with the analysis of clinical samples, SIX1 expression was generally upregulated in NSCLC cells compared with HBE. The endogenous expression of SIX1 was the highest in PC-9 cells, whereas A549 and H1299 cells harbored lower SIX1 protein and mRNA level (Fig. 3A). Therefore, the lentivirus overexpressing SIX1 was transfected into A549 and H1299 cells, while the siRNA knocking down SIX1 was applied to PC-9 cells. The structure of the lentiviral vector overexpressing SIX1 and its transfection efficiency in NSCLC cell lines were shown in Fig. S1. We found that SIX1 was efficiently overexpressed in A549 and H1299 cells and inhibited in PC-9 cells, and validated by western blotting, RT-PCR and immunofluorescence staining (Fig. 3B-D, Fig. S2). We proceeded to evaluate the effect of SIX1 on NSCLC cells viability by EdU assays. The data showed that overexpression of SIX1 contributed to more EdU positive cells, while knockdown of SIX1 exhibited an opposite effect (Fig. 3E). Additionally, CCK-8 assays also showed an increased cell growth in SIX1-overexpressing NSCLC cells, while knockdown of SIX1 diminished cell proliferation *in vitro* (Fig. 3F-H). Collectively, the above results demonstrated that SIX1 could positively regulate the proliferation of NSCLC cells.

### SIX1 contributed to migration, invasion and EMT of NSCLC cells

**Figure 4:**
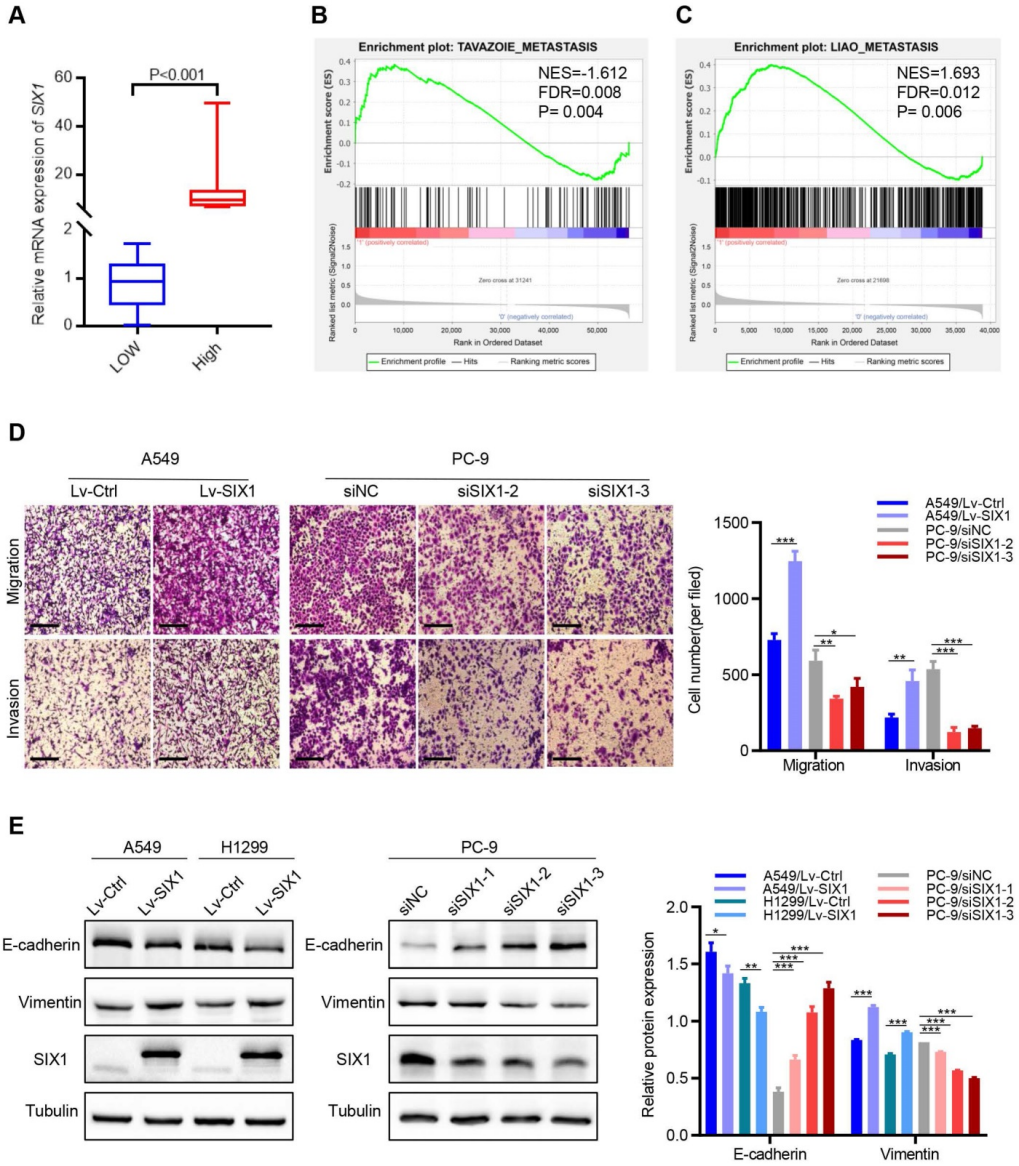
** SIX1 induced EMT and promoted migration and invasion.** (A)* SIX1* expression in the high-*SIX1* sample compared with the low-*SIX1* counterparts of the TCGA lung cancer database. (B, C) *SIX1* expression was positively correlated with metastasis-related genes in NSCLC by GSEA. (D) Cell migration and invasion assays of indicated cells by transwell chamber. (E) The expression of E-cadherin and Vimentin was changed after up- or down- regulation of SIX1 in NSCLC cells. *p< 0.05, **p < 0.01, ***p < 0.001. Graphs show mean ± SD.

The above database analysis showed that *SIX1* was elevated in metastatic SKCM tissues than in primary SKCM tumor tissues. In addition, upregulated SIX1 might be associated with late stage in NSCLC. Therefore, we next investigated the effect of SIX1 on metastasis in NSCLC cells. GSEA was performed based on RNA-sequencing data from the TCGA lung cancer database. The samples were divided into the *SIX1*-low group and *SIX1*-high group according to the quartile of *SIX1* expression (Fig. 4A, SIX1-low: Q1, SIX1-high: Q4). GSEA revealed that the gene sets relevant to metastasis were enriched in samples with high *SIX1* expression (Fig. 4B and 4C). Next, we conducted transwell and wound healing experiments to investigate the effects of SIX1 on cell migration and invasion in NSCLC *in vitro*. Evidently, A549 cells with SIX1 overexpression exhibited stronger migration and invasive abilities compared with control cells, while knockdown of SIX1 dramatically inhibited migration and invasion in PC-9 cells (Fig. 4D, Fig. S3).

EMT has been reported to be critical for the increased cellular motility and invasiveness. Therefore, we further investigated the potential effect of SIX1 on EMT processes in NSCLC. We found that the protein levels of epithelial marker (E-cadherin) were notably decreased, and the level of a mesenchymal marker (Vimentin) was significantly increased in A549 and H1299 cells with SIX1 overexpression. Conversely, knockdown of SIX1 induced the up-regulation of E-cadherin and down-regulation of Vimentin in PC-9 cells (Fig. 4E). Similar results were found in the immunofluorescence assay of Vimentin (Fig. S4). In conclusion, the results confirmed that SIX1 could promote the migration, invasion, and EMT process of NSCLC cells.

### SIX1 activated the Notch signaling pathway in NSCLC cells

**Figure 5:**
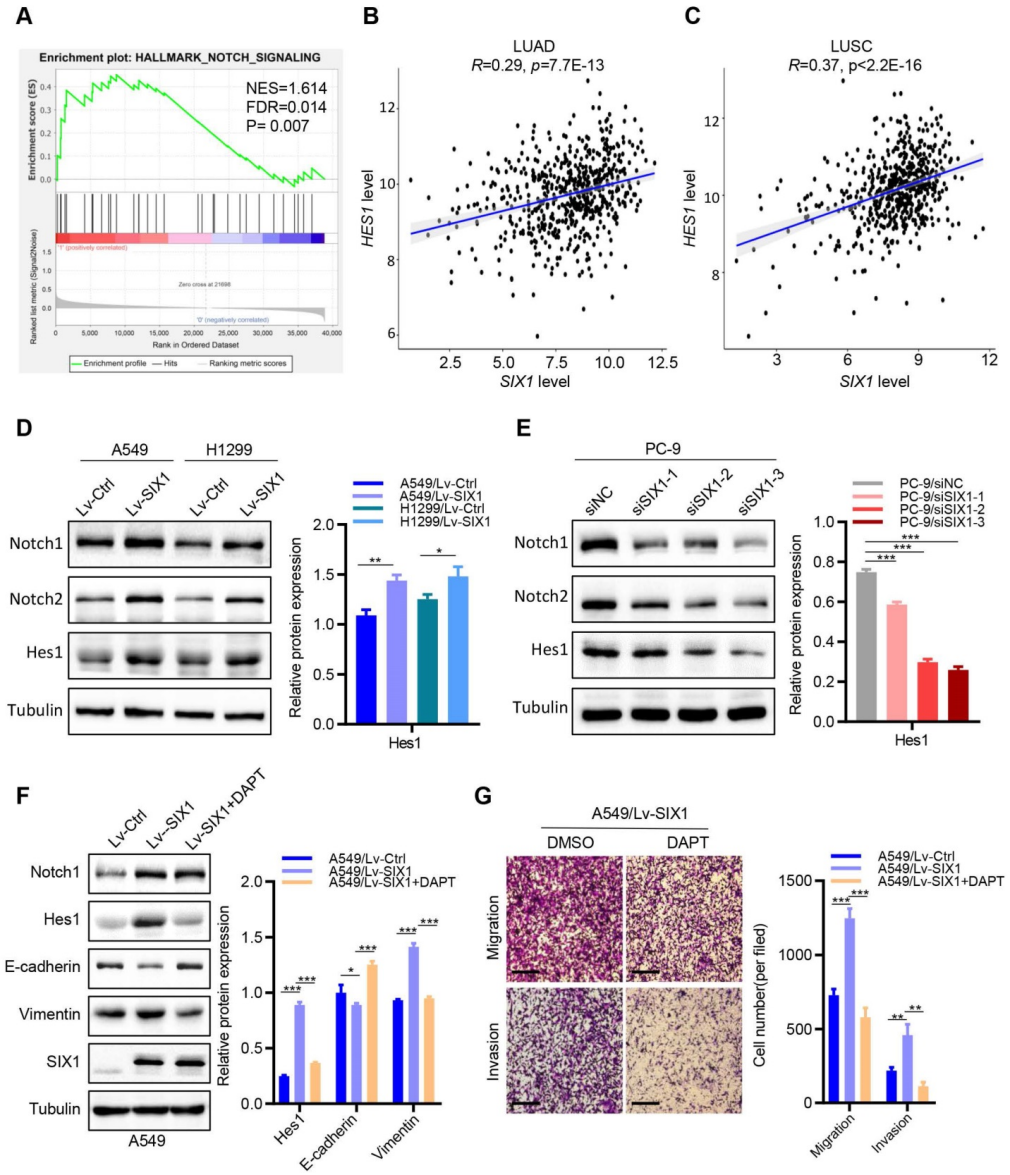
** SIX1 activates the Notch signaling pathway in NSCLC cells.** (A) The *SIX1* expression was positively correlated with the Notch signaling pathway in NSCLC by GSEA. (B) The *SIX1* and* HES1* mRNA expression was positively correlated in TCGA LUAD samples. (C) The *SIX1* and* HES1* mRNA expression was positively correlated in TCGA LUSC samples. (D) (E) The protein level of Notch1, Notch2 and Hes1 in indicated cells. (F) The protein level of Notch1, Notch2, Hes1 E-cadherin, Vimentin and SIX1 upon treated with Notch pathway inhibitor (DAPT, 10uM). (G) Cell migration and invasion assays of indicated cells with or without the DAPT treatment by transwell chamber. *p< 0.05, **p < 0.01, ***p < 0.001. Graphs show mean ± SD.

Among the various pathways revealed by GSEA enrichment analysis, we observed that the Notch signaling pathway was positively correlated with the expression of *SIX1* in NSCLC (Fig. 5A). Previous studies have demonstrated that the activated Notch signaling pathway played critical roles in regulating multiple malignant biological behaviors in NSCLC. Therefore, we tested whether SIX1 might exert its biological functions by activating the Notch signaling pathway. We first detected the correlation between SIX1 and the Notch pathway target gene, Hes1, in the TCGA database. We found that *SIX1* and *HES1* mRNA expressions were positively correlated in both LUAD and LUSC (Fig. 5B and 5C). Furthermore, overexpression of SIX1 robustly increased the protein levels of Notch1, Notch2 and Hes1 in A549 and H1299 cells (Fig. 5D), but without significant change of *Notch1* and *Notch2* mRNA levels, despite an increase in the expression of *Hes1* mRNA (Fig. S5). In contrast, knockdown of SIX1 in PC-9 cells remarkably decreased the protein levels of Notch1, Notch2, and Hes1 (Fig. 5E). However, when Notch1 was knocked down, EMT was notably reversed in A549 cells, while the expression of SIX1 did not change (Fig. S6). These results suggested that SIX1 might act as an upstream to activate the Notch signaling pathway in NSCLC cells.

Based on the above results that overexpression of SIX1 could promote proliferation, migration, invasion, and EMT processes of NSCLC cells *in vitro*, we next investigated the effects of the Notch signaling inhibitor (a γ-secretase inhibitor, DAPT) 17 on SIX1-induced malignant biological behaviors. Interestingly, clonal formation analysis showed that DAPT significantly reduced SIX1-induced proliferation in A549 cells (Fig. S7). In addition, DAPT drastically attenuated the increased expression of Hes1 and Vimentin caused by the upregulation of SIX1, while the E-cadherin expression was elevated. Similarly, DAPT had no significant effect on the expression of SIX1. Furthermore, the abilities of cell migration and invasion were measured by transwell assays. We found that cell migration and invasion were weakened in the LV-SIX1 +DAPT group compared with the Lv-SIX1 group (Fig. 5F and 5G). Collectively, overexpression of SIX1 could promote the activation of the Notch signaling pathway, while inhibition of the Notch signaling could reduce the carcinogenic effects caused by SIX1 in NSCLC.

### SIX1 facilitated tumor growth of NSCLC cells *in vivo*

**Figure 6:**
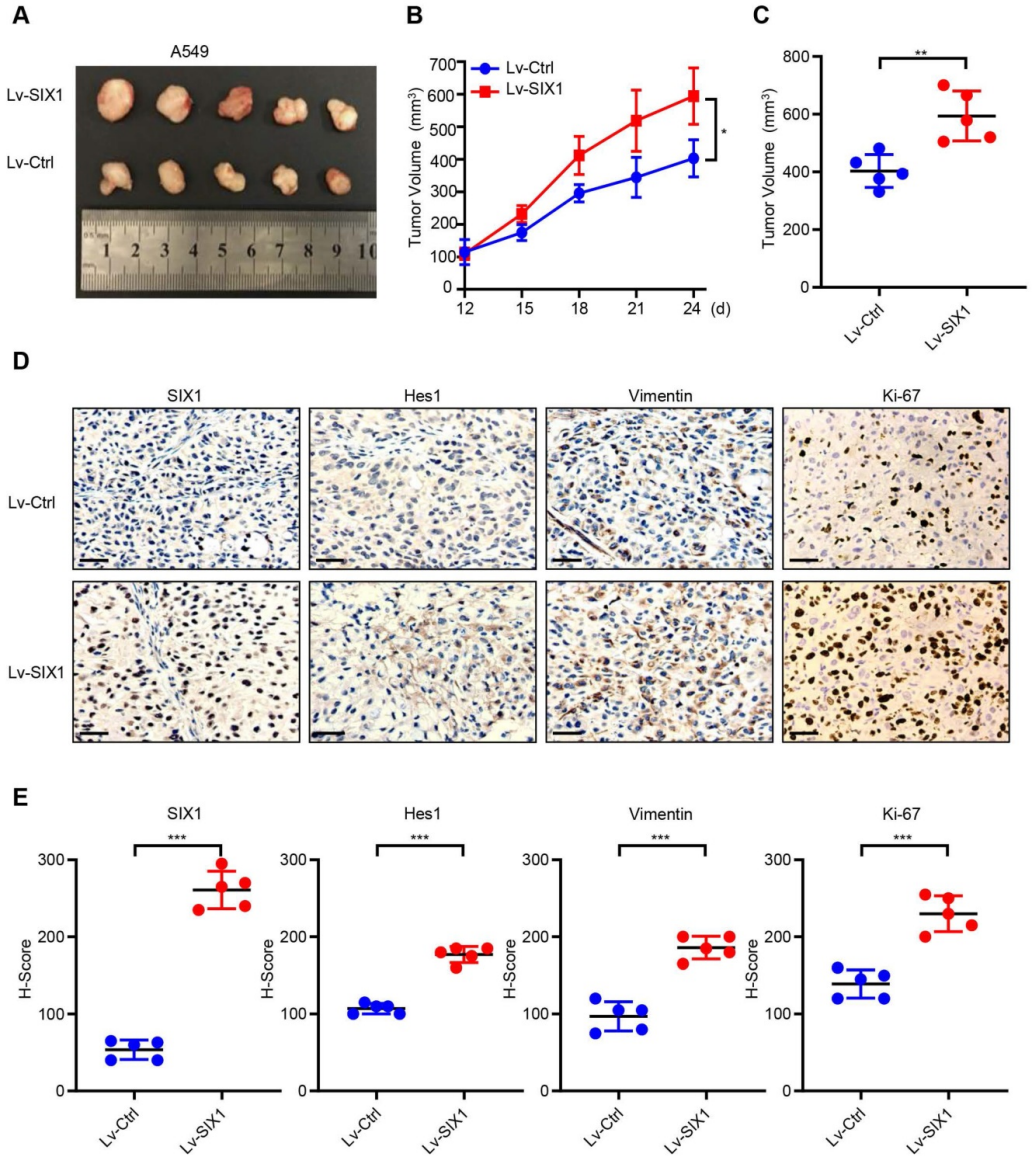
** SIX1 facilitates the growth of A549 cells *in vivo.*** (A) Tumor xenografts of each group. n=5. (B) Tumor growth curve in each group. (C) Tumor volumes on day 24 were shown for groups containing 5 mice each. (D) (E) IHC staining was performed to determine the expression of SIX1, Hes1, Vimentin and Ki-67 in the tumor. *p< 0.05, **p < 0.01, ***p < 0.001. Graphs show mean ± SD.

To further investigate the potential oncogenic effects of SIX1, we generated the subcutaneous tumor by injecting A549/Lv-Ctrl cells and A549/Lv-SIX1 cells (5×10^6^ cells/mouse) in BALB/c nude mice. The tumor size was measured from day 12 and recorded every three days, and a growth curve was plotted. Tumor tissue was removed from both groups on day 24, the endpoint of observation, and the mean tumor size was quantified. The results showed that overexpression of SIX1 significantly facilitated tumor growth in *vivo* (*p*< 0.05, Fig. 6A-C). We further performed IHC staining of tumor tissues on both groups. Data confirmed that SIX1 was overexpressed in the Lv-SIX1 tumor tissues compared with the control group, mainly located at the nucleus. Importantly, Hes1, Vimentin, and Ki-67 levels were also higher than those in the control groups (Fig. 6D and 6E). Therefore, these findings provide evidence that SIX1 could also act as an oncogene that induced Notch signaling activation and promote tumor growth and EMT process *in vivo.*

## Discussion

Aberrant activation of embryonic developmental pathways is a pervasive tumor-promoting mechanism. Previous studies have indicated that SIX1, a transcription factor involved in embryonic development, is frequently dysregulated in numerous human cancers, and its increased expression is associated with poor clinical outcomes 18. Usually, SIX1 is highly expressed during embryogenesis and rarely expressed in normal adult tissues 19. SIX1 is expressed in the distal epithelium and stroma of lung branching airways, and its absence can lead to differentiation of lung stroma and fibroblasts, leading to defective development of lung septum 20. In studies on NSCLC, although the upregulation of SIX1 has been observed to promote preinvasive-to-invasive LUAD progression 15, the expression status, prognostic value, and biological function of SIX1 in NSCLC remain to be further studied.

Consistent with previous studies, we also confirmed that the expression of SIX1 in NSCLC tissues was higher than that in normal tissues through the analysis of multiple databases 21, 22. However, previous studies yielded inconsistent results regarding the prognostic value of SIX1 in NSCLC. Liu Q et al. reported that the higher expression of SIX1 was associated with the greater possibility of the tumorigenesis, but it had no correlation with node metastasis, TNM stage and OS 21. A previous study conducted by Xia et al. showed that the expression of *SIX1* was associated with large tumor size, advanced tumor stage, and distant metastasis of NSCLC 22. Moreover, Mimae T et al. showed that the upregulation of SIX1 could contribute to preinvasive-to-invasive transition in ADC 15. In our study, we did not find a significant correlation between the SIX1 expression and lymph node metastasis or TNM stage in TCGA database. However, our data mining based on the GSE31210 showed that the high SIX1 expression was associated with advanced tumor stage and a high level of SIX1 was correlated with shortened time to relapse and decreased OS in patients with NSCLC. The possible explanation was the difference in populations of patients and the difference in the cut-off values in different studies. The samples of the study of Xia et al., Mimae T et al, and GSE31210 were from Asian populations, while most samples included in the TCGA database came from American population. Furthermore, we divided sample into the SIX1-low group and SIX1-high group according to the quartile of SIX1 expression rather than the median expression. Recently, several studies have confirmed DACH1 functions as tumor suppressor in NSCLC and many other cancers 23-25. In addition, a recent study in breast cancer showed that a combination of SIX1 with DACH1 could effectively distinct patients with high relapse risk within 3, 5, and 8 years 26. Although SIX1 might serve as a promising biomarker for NSCLC prognosis to some extent, a more comprehensive predictive model is helpful to predict the prognosis of patients with NSCLC.

Accumulating evidence has suggested that SIX1 was involved in tumor initiation and progression by regulating multiple activities of cancer cells. SIX1 is known to be essential for cell growth and proliferation by enhancing the transcription of a range of target genes. For example, SIX1 was overexpressed in breast cancer and led to increased cell proliferation by upregulating cyclin A1 9, 27. H. Ford et al. proposed that SIX1 was involved in the control of the G2/M transition of the cell cycle in the breast cancer cell, thereby enhancing the tumorigenic potential 28. SIX1 activated the cyclin D1 and c-Myc to control cell proliferation, survival, and motility in rhabdomyosarcoma 29. SIX1 could promote proliferation via upregulating the connective tissue growth factor (CTGF) in glioblastoma cells 30. Additionally, recent studies have shown that SIX1 directly increases the transcription of glycolysis genes and enhances the glycolysis of cancer cells to promote tumor growth 31. Similarly, we found that overexpression of SIX1 could promote the proliferation and tumor growth of NSCLC, while knockdown of SIX1 exhibited the opposite effect. Therefore, SIX1 might be a potential target for controlling the growth of NSCLC.

Distant metastasis was a significant challenge in the battle against cancers. Previous studies have indicated that EMT, a reversible dynamic process in which epithelial cells lose the intercellular adhesion and transit into aggressive mesenchymal cells, could enhance the motility and invasiveness of cancer cells, thus promoting the metastasis and progression of tumors 32, 33. EMT was usually characterized by the downregulation of epithelial cell markers (E-cadherin, Desmoplakin, and Occludin) and upregulation of mesenchymal cell markers (Vimentin, N-cadherin, and Fibronectin) 34. SIX1-induced EMT has been observed in many human cancers. SIX1 induced human mammary carcinoma cells to undergo EMT and metastasis through TGF-β signaling 35. SIX1 promotes EMT in colorectal cancer through ZEB1 activation 36. Zhu et al. showed that SIX1 could transcriptionally activate the Vimentin, thereby promoting gastric cancer cell migration and invasion 37. Here, we found that SIX1 promoted the migration and invasion of NSCLC cells. Upregulated SIX1 expression showed an increased expression of Vimentin and a decreased expression of E-cadherin, while downregulated SIX1 resulted in the opposite result. These data demonstrated that SIX1 might promote NSCLC progression by inducing EMT.

Notch signaling is an evolutionarily conserved pathway that is critical for the development and homeostasis in various tissues 38. Previous studies have also indicated that the Notch signaling pathway was involved in carcinogenesis and tumor progression in numerous malignant cancers by regulating cell proliferation, differentiation, and apoptosis 39. In addition, the Notch signaling played an essential role in the occurrence of lung cancer and cross-talked with multiple transcriptional factors to trigger EMT, thereby promoting the progression of NSCLC 40. In this study, we found that the Notch signaling pathway was significantly enriched in the group with high SIX1 expression, and the level of* HES1,* the downstream target gene of Notch signaling, was positively correlated with *SIX1* in NSCLC. Further studies showed that SIX1 could activate the Notch signaling and its downstream oncogenes. Importantly, SIX1-driven malignant biological behaviors could be blocked by the Notch pathway inhibitor DAPT in NSCLC cells. In summary, we demonstrated that SIX1 might promote NSCLC progression by activating the Notch signaling pathway. Given that transcription factors are notoriously difficult to target directly, our results suggested that inhibition of Notch signaling by a γ-secretase inhibitor (DAPT) might be a promising therapeutic strategy for SIX1-driven NSCLC patients in the future.

However, there were still some limitations in our research. First, it has been reported that the gain-of-function mutations and amplification of SIX1 enhanced its ability to promote tumor growth 41, 42. Therefore, more studies are needed to investigate the mechanisms leading to the SIX1 re-expression in NSCLC. Second, we found that overexpression of SIX1 induced an increase in Notch1, Notch2, and Hes1 protein levels without significant change of *Notch1* and *Notch2* mRNA levels, despite an increase in the expression of *Hes1* mRNA. These results indicated that SIX1 might be involved in the post-transcriptional regulation of the Notch receptor, and further studies are needed to explore the underlying mechanism. Third, additional oncogenic signaling pathways regulated by SIX1 need to be investigated in NSCLC, which is essential for a comprehensive understanding of SIX1's role in NSCLC and the development of appropriately targeted drugs in NSCLC.

In conclusion, we demonstrated that *SIX1* was markedly upregulated in tumor tissues than the corresponding adjacent normal tissues in NSCLC. Elevated SIX1 expression was correlated with poor prognosis. Overexpression of SIX1 in NSCLC contributed to cell proliferation, migration, invasion, and the dynamic properties of EMT*.* Moreover, we found that SIX1 could activate the Notch signaling pathway in NSCLC, and inhibition of the Notch signaling pathway with γ-secretase inhibitor could reverse a series of SIX1-mediated malignant phenotypes. Overall, our findings suggested that SIX1 might serve as a prognostic biomarker for NSCLC and highlighted the potential value of the SIX1/Notch axis as a promising target for combating NSCLC progression.

## Supplementary Material

Supplementary figures.

## Figures and Tables

**Table 1:** The sequences of primers used for experiments in this study.

Primer	Sequence 5'-3'
*SIX1* Forward Primer	CTGCCGTCGTTTGGCTTTAC
*SIX1* Reverse Primer	GCTCTCGTTCTTGTGCAGGT
*β-actin* Forward Primer	CATGTACGTTGCTATCCAGGC
*β-actin* Reverse Primer	CTCCTTAATGTCACGCACGAT
*Notch1* Forward Primer	GAGGCGTGGCAGACTATGC
*Notch1* Reverse Primer	CTTGTACTCCGTCAGCGTGA
*Notch2* Forward Primer	CCTTCCACTGTGAGTGTCTGA
*Notch2* Reverse Primer	AGGTAGCATCATTCTGGCAGG
*Hes1* Forward Primer	TCAACACGACACCGGATAAAC
*Hes1* Reverse Primer	GCCGCGAGCTATCTTTCTTCA
*E-cadherin* Forward Primer	CGAGAGCTACACGTTCACGG
*E-cadherin* Reverse Primer	GGGTGTCGAGGGAAAAATAGG
*Vimentin* Forward Primer	GACGCCATCAACACCGAGTT
*Vimentin* Reverse Primer	CTTTGTCGTTGGTTAGCTGGT

## Abbreviations

SIX1: Sine oculis homeobox homolog 1
NSCLC: non-small cell lung cancer
GSEA: Gene set enrichment analysis
EMT: epithelial-mesenchymal transition
LUAD: lung adenocarcinoma
LUSC: lung squamous cell carcinoma
LCLC: large cell lung cancer
FBS: fetal bovine serum
IHC: immunohistochemistry
