# Supplementary material for: SIX1 Predicts Poor Prognosis and Facilitates the Progression of Non-small Lung Cancer via Activating the Notch Signaling Pathway
Source: J Cancer. 2022 Jan 1;13(2):527–40. doi: 10.7150/jca.61385 (PMC8771509; doi:10.7150/jca.61385)
Supplement: Supplementary file 1 — Supplementary figures. [file jcav13p0527s1.pdf]

Supplementary Figures

Supplementary Figure S1

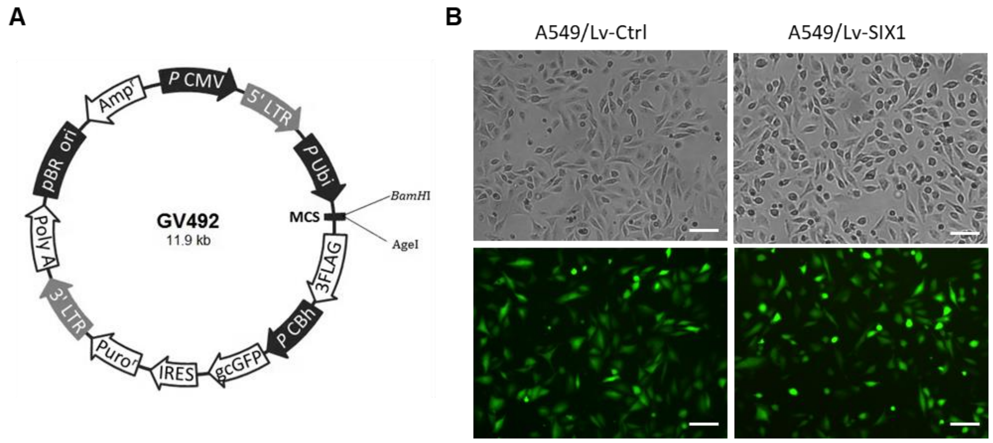

Supplementary Figure S1. The structure of a lentiviral vector overexpressing SIX1 and its transfection efficiency in NSCLC cell lines. (A) Structure of the lentiviral vector of overexpressing SIX1. (B) Green fluorescent protein (GFP) image showing the transfection efficiency of A549 cells.

Supplementary Figure S2

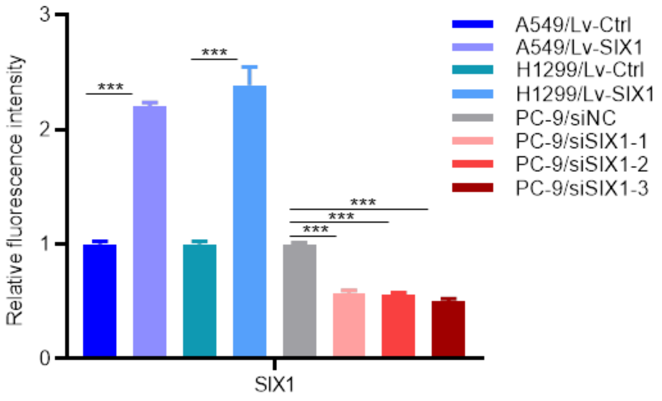

Supplementary Figure S2. Quantification of relative fluorescence intensity of SIX1 staining.

Supplementary Figure S3

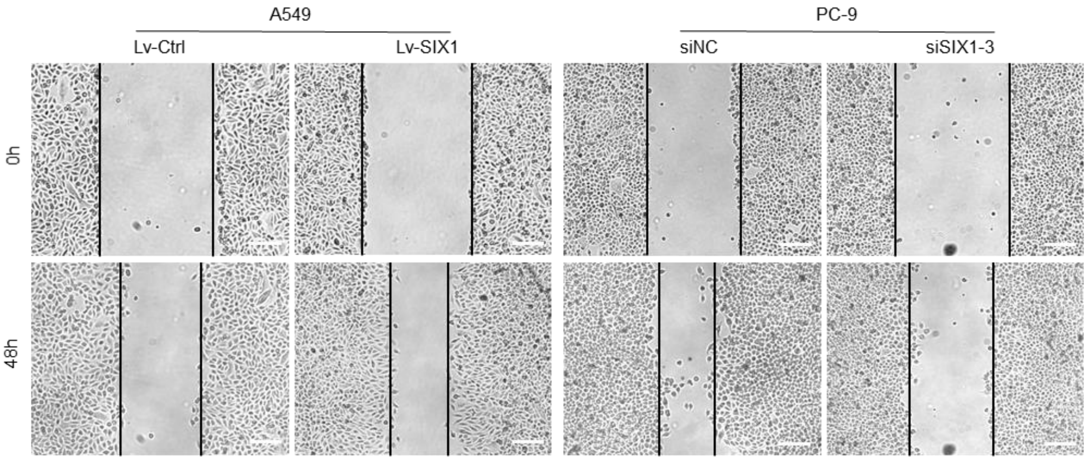

Supplementary Figure S3. Wound healing assays the effect of SIX1 overexpression and knockdown on cell migration.

Supplementary Figure S4.

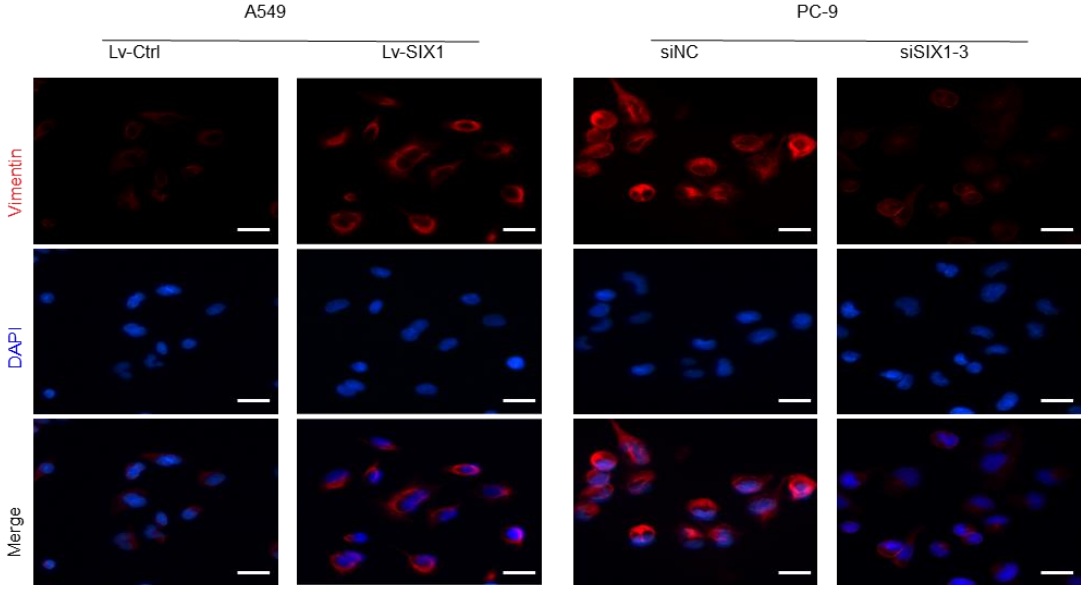

Supplementary Figure S4. Immunofluorescence images of Vimentin in the indicated cells.

Supplementary Figure S5

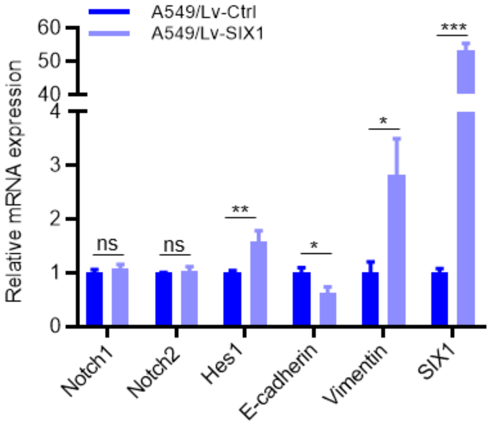

Supplementary Figure S5. The indicated mRNA levels in NSCLC cells upon overexpressing SIX1.

Supplementary Figure S6

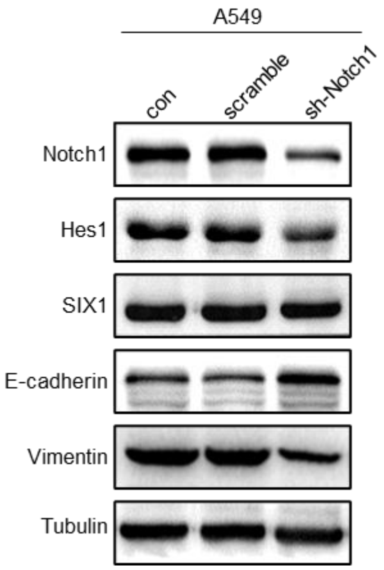

Supplementary Figure S6. The indicated protein levels in NSCLC cells upon knocking down of Notch1.

Supplementary Figure S7

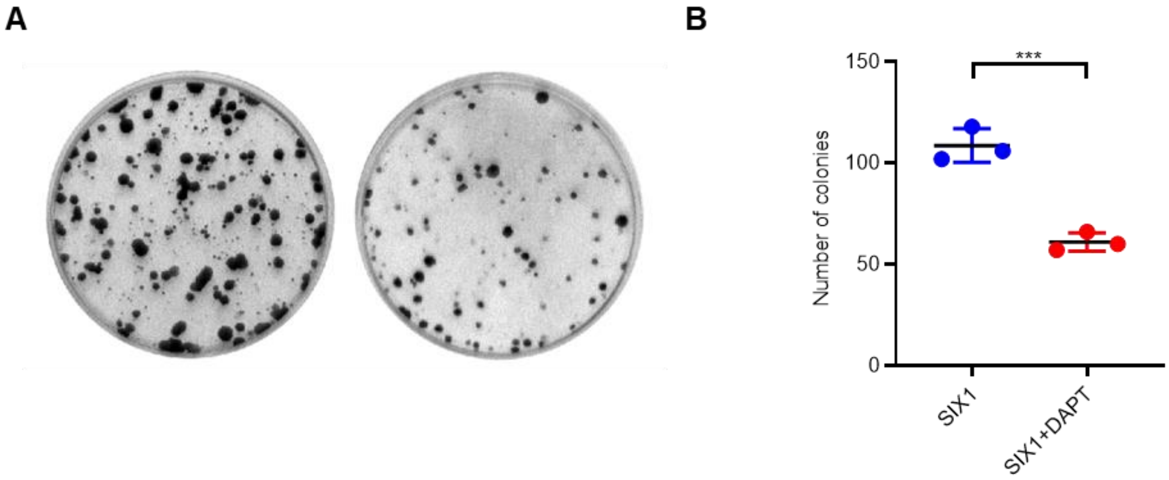

Supplementary Figure S7. Clonal formation analysis the cell growth upon treatment with DAPT.
